# The 60S ribosomal protein L13 is the most preferable reference gene to investigate gene expression in selected organs from turkeys and chickens, in context of different infection models

**DOI:** 10.1186/s13567-016-0388-z

**Published:** 2016-10-20

**Authors:** Taniya Mitra, Ivana Bilic, Michael Hess, Dieter Liebhart

**Affiliations:** 1 Department for Farm Animals and Veterinary Public Health, Clinic for Poultry and Fish Medicine, University of Veterinary Medicine Vienna, Veterinärplatz 1, 1210, Vienna, Austria; 2 Christian Doppler Laboratory for Innovative Poultry Vaccines (IPOV), University of Veterinary Medicine Vienna, Veterinärplatz 1, 1210, Vienna, Austria

## Abstract

Evaluation of reference genes for expression studies in chickens and turkeys is very much limited and unavailable for various infectious models. In this study, eight candidate reference genes HMBS, HPRT1, TBP, VIM, TFRC, RPLP0, RPL13 and RPS7 were evaluated by five different algorithms (GeNorm, NormFinder, BestKeeper©, delta CT, RefFinder) to assess their stability. In order to analyze a broad variation of tissues, spleen, liver, caecum and caecal tonsil of different aged specific pathogen free (SPF) layer chickens and commercial turkeys, uninfected or infected with the extracellular pathogen *Histomonas meleagridis*, were included. For tissue samples from SPF chickens RPL13 and TBP were found to be the most stable reference genes. Further testing of RPL13 and TBP in the same organs of uninfected and infected SPF broiler chickens with the intracellular pathogen fowl aviadenovirus confirmed this finding. In tissue samples from turkeys, a stable expression of RPL13 and TFRC genes was noticed. Overall, the determined reference genes should be considered whenever gene expression studies in spleen, liver, caecum and caecal tonsil of chickens and turkeys are performed.

**Electronic supplementary material:**

The online version of this article (doi:10.1186/s13567-016-0388-z) contains supplementary material, which is available to authorized users.

## Introduction

Gene expression analysis provides insights into complex biological regulatory processes and has become an essential part in various molecular biology studies. Reverse transcription quantitative real-time polymerase chain reaction (RT-qPCR) is, in many studies, the method of choice for the detection and quantification of mRNA [1]. However, this method can be affected by technical variations in template quantity, quality, reverse transcription process and data analysis which impede correct measurements of true biological deviations [2, 3]. It is therefore essential to normalize these variations. There are several methods to eliminate technically induced variations from the true biological diversity such as in situ calibration, generic normalization against total mRNA, measuring DNA content of total nucleic acid or normalization with validated reference genes [4]. According to minimum information for publication of quantitative real-time pcr experiments (MIQE) guidelines, a reliable method of normalization uses reference genes which should be validated for every species and also on the basis of different experimental treatments [5]. The use of a single reference gene is considered to be an improper approach for gene expression studies, and the application of several genes for normalization is highly recommended to avoid erroneous results introduced by technical manipulation of samples [4, 6]. In recent years, reference genes were established for different animal species, such as cattle, pig, sheep, goat, horse and fish [7–12]. Also in chickens (*Gallus gallus*), a number of studies validated reference genes under different physiological conditions [13–20]. However, the assessment of genes used for normalization of gene expression during infection with an extracellular pathogen is completely lacking. Furthermore, only a single study evaluated reference genes for brain tissue in turkeys (*Meleagris gallopavo*) [18]. This prompted us to validate reference genes in spleen, liver, caecum and caecal tonsils from healthy and infected SPF layer chickens and turkeys with the extracellular pathogen *Histomonas meleagridis* at different ages. In SPF broiler chickens, preselected reference genes were further evaluated using samples from birds infected with the intracellular pathogen fowl aviadenovirus (FAdV).

## Materials and methods

### Sample selection

A total of 252 different tissue samples from 27 SPF layer-type chickens (VALO, BioMedia, GmBH, Osterholz-Scharmbeck, Germany), 12 SPF broiler chickens (Animal Health Service, Deventer, Netherlands) and 24 commercial turkeys (B.U.T.6™; Aviagen Turkeys Ltd, Tattenhall, UK) were included in the analysis (Table 1). Samples from spleen, liver, caecum and caecal tonsil were collected from three non-infected SPF layer-type chickens and three turkeys at six time points between their first and their 49^th^ day of life, to cover age related changes in gene expression. In addition, the effect of infectious pathogens on the expression of reference genes in host birds was investigated by collecting samples during the course of disease. For that, the same organs were sampled on three different time points from SPF layer-type chickens and on two time points from turkeys following infection with the extracellular pathogen *H. meleagridis* between the 35^th^ and 49^th^ day of life. Infected birds showed inflammation of the caeca and the livers with the exception of chickens sampled on the day 49, when two out of three birds had no lesions. Healthy SPF broiler chickens were sacrificed at the age of 4, 7 and 21 days of life to collect spleen, liver, caecum, and caecal tonsils of three birds each time point. The same organs of three additional SPF broiler chickens that were infected with the intracellular pathogen fowl aviadenovirus were sampled at day 7 of life. Swollen marble-like livers with a colour ranging from yellow to brown were observed in infected birds [21].

**Table 1:** **Organ samples used in this study**

Animal type/species	Day of life								
	1	4	7	14	21	28	35	38	49
Healthy SPF layer chickens	x	n.d.	n.d.	x	n.d.	x	x	x	x
*H. meleagridis*-infected SPF layer chickens	n.d.	n.d.	n.d.	n.d.	n.d.	n.d.	x	x	x
Healthy turkeys	x	n.d.	n.d.	x	n.d.	x	x	x	x
*H. meleagridis*-infected turkeys	n.d.	n.d.	n.d.	n.d.	n.d.	n.d.	x	x	n.d.
Healthy SPF broiler chickens	n.d.	x	x	n.d.	x	n.d.	n.d.	n.d.	n.d.
Fowl aviadenovirus-infected SPF broiler chickens	n.d.	n.d.	x	n.d.	n.d.	n.d.	n.d.	n.d.	n.d.

From different groups, spleen, liver, caecum and caecal tonsil were sampled in between 1^st^ and 49^th^ day of life.

x: sampling of three birds.

n.d.: not done.

All samples were collected separately during post mortem investigations directly after euthanization of birds and stored in RNA*later* RNA stabilization reagent (Qiagen, Hilden, Germany) at −80 °C.

### Gene selection

Based on findings from previous gene expression studies on different bird tissues [13, 14, 17, 18], eight genes—HMBS, HPRT1, TBP, VIM, TFRC, RPLP0, RPL13 and RPS7, known to be involved in different basic metabolic and structural pathways were selected to be tested for their suitability as reference genes (Table 2). Analyses of these genes were performed at the time points mentioned above in spleen, liver, caecum and caecal tonsil samples from SPF layer chickens and commercial turkeys. Samples from healthy birds together with those from birds infected with *H. meleagridis* were processed. Further analyses of RPL13 and TBP were performed using the same organs from healthy and fowl aviadenovirus infected SPF broiler chickens.

**Table 2:** **Details of selected potential reference genes**

Gene/symbol	Protein	Physiological functions	Accession number for chicken	Accession number for turkey	Primer and probe sequences (F-forward primer; R-reverse primer; P-probe)
TFRC	Transferrin receptor protein	Cellular uptake of iron	NM_205256.2	XM_003209136.2	F:AGCTGTGGGTGCTACTGAA R:GGCAGAAATCTTGACATGG P:HEX-CTCTGCCATGCTGCATGCCA-BHQ1
TBP	Binding protein TATA box	Transcription factor	NM_205103.1	XM_010707033.1	F:CTGGGATAGTGCCACAGCTA R:GCACGAAGTGCAATGGTTT P:ROX-TGCAACCAAGATTCACCGTGGA-BHQ2
HPRT1	Hypoxanthine–guanine phosphoribosyl-transferase I	Enzyme in the purine pathway	NM_204848.1	XM_010715191.1	F:GCTCATCATGGACAGGACAG R:CACAGAGAGCTACAATGTGGTG P:CY5-TGCCCTTCATAATTTCACGTGCCA-BHQ3
VIM	Vimentin	Cytoskeletal component responsible for maintaining cell integrity	NM_001048076.1	XM_010712706.1	F-TGAGTCCCTGCAAGAAGAAA R:CAGGGCAGCAGTAAGATCAG P:ROX-TCCGGGAACTGCAGGCTCAA-BHQ2
RPS7	40S acidic ribosomal protein S7	Small ribosomal subunit	XM_004940516.1	NM_001285787.1	F-TGGTATATCCCAGGCTCTCC R-TCAGCTGAGGAACTGGTACG P:FAM-TCAACTCCCGCAGCTGAGCC-TAM
RPL13	60S ribosomal protein L13	Large ribosomal subunit	NM_204999.1	XM_010718177.1	F:GGAGGAGAAGAACTTCAAGGC R:CCAAAGAGACGAGCGTTTG P:HEX-CTTTGCCAGCCTGCGCATG-BHQ1
HMBS	Hydroxymethyl-bilane synthase	Production of heme	XM_417846.5	XM_010723546.1	F:CCTGCCAACTTCTCTTCCTC R:CAACAGCATCAAGTGGGTTC P:FAM-TGCAAATAGCACCAATGGTAAAGCCA-TAM
RPLP0	60S acidic ribosomal protein P0	Large ribosomal subunit	NM_204987.2	XM_003211079.2	F-CAATGGCAGCATTTACAACC R:CAGGAAACGCTTGTGCAG P:CY5-TCTCCTCAGTGATGTCCAGCACTTCA-BHQ3

### Total RNA extraction and analysis for purity and integrity

Total RNA was prepared from RNA*later* ^®^ (Qiagen) stabilized liver, spleen, caecum and caecal tonsil tissues. Tissue samples were homogenised separately using QIAshredders (Qiagen) and total RNA was extracted by RNeasy^®^ mini kit (Qiagen) according to manufacturer’s instructions and RNA was stored at −80 °C before further use. Every sample was assessed according to following criteria: the nucleic acid purity was analyzed with A260/280 and additionally A260/230 ratio by NanoDrop 2000 (ThermoFisher scientific, Vienna, Austria) to ascertain that the RNA was free from contaminates like guanidine, glycogen and EDTA. The RNA quality and quantity of every sample was further surveyed by chip-based capillary electrophoresis Bioanalyzer 2100 (Agilent technologies, Waldbronn, Germany). In this process, the RNA concentration and the integrity of total RNA together with the presence or absence of degradation products were estimated by measuring the entire electrophoretic trace of each sample, given by the RNA integrity number (RIN) [22].

### RT-qPCR

An identical set of primer and probe sequences were designed for both poultry species, chickens and turkeys, to target highly conserved regions of reference genes. Primers and probes were selected by using the respective sequence information from the NCBI database (Table 2) and GenScript real-time PCR (TaqMan) primer design software with default settings. One step real-time quantitative reverse transcription polymerase chain reaction (RT-qPCR) used TaqMan chemistry and Brilliant III Ultra-Fast QRT-PCR master mix kit (Agilent technologies). Amplification and quantification of reference genes mRNA were performed using AriaMx real-time PCR system (Agilent Technologies) together with the Agilent AriaMx1.0 software (Agilent Technologies). Thermal cycle profile for RT-qPCR was adjusted as follows: 1 cycle of reverse transcription at 50 °C for 10 min followed by 95 °C for 3 min to hot start, 40 cycles of amplification at 95 °C for 5 s and 60 °C for 10 s. Concentrations of 200–900 nM for primers and 100 nM for probes were ascertained by tenfold serial dilutions of RNA (100, 10, 1, 0.1 ng) to determine the optimal primer concentration and the highest efficiency of RT-qPCR reactions. Further details on the selected primer concentrations and the efficiency values are given in the Additional file 1. All samples were analysed in duplicate and different types of controls such as NRT (non reverse transcriptase) and NTC (non template control) were run with every plate to determine possible genomic DNA contamination and overall PCR contamination. The mean CT value of each duplicate was further used for the gene expression analysis. Overall, the RT-qPCR investigation complies with the MIQE guidelines [5].

### Gene expression analysis

Organ samples of healthy and infected SPF layer chickens and turkeys were analyzed separately. The stability of gene expression was determined by calculations using different software algorithms: GeNorm, NormFinder, BestKeeper© and delta CT. In addition, RefFinder was used to rank the genes on the basis of stability from the most to the least stable reference genes.

### GeNorm

The GeNorm algorithm was used to calculate the average of pairwise variation of one gene with all the other potential reference genes and to identify their average expression stability (M). The gene with lowest M value was assumed to be the highest stable gene [23]. Genes with a threshold of 1.5 were considered stable whereas a threshold of 1.0 and below characterized the most stable genes [17].

### NormFinder

NormFinder software generates a stability measure (Sv) which indicates an increased stability in gene expression by a low value. NormFinder software allows direct estimation of expression variation between different organ groups and ranked genes according to the similarity of their expression profiles by using a model-based approach. The chance to introduce systemic errors in genes with a low Sv value were found to be marginal, as previously described [24].

### BestKeeper©

BestKeeper© calculated the standard deviation (SD) based on raw crossing point (CP) including the inter-gene relationship with the help of Pearson correlation coefficient matrix. Highly correlated genes were combined into an index which is called BestKeeper© index. The comparison of the correlation of each gene with BestKeeper© index gave a correlation coefficient value (r) with the probability value (*p*), as explained earlier [25]. BestKeeper© calculated the most stable gene by the lowest coefficient of variance (r) and standard deviation (SD).

### Delta CT

Delta CT method was used to compare CT values of all possible gene combinations. An increased or decreased level of deviation in delta CT pattern is formed by comparing the possible gene combinations. Least amount of deviation means least amount variability of gene expression within the samples [26].

### RefFinder

RefFinder is a web-based tool that integrates the current major computational programs, including GeNorm, Normfinder, BestKeeper©, and the delta CT method, to compare and rank the stability of the investigated candidate reference genes. Based on the rankings from each program, RefFinder assigns an appropriate value to an individual gene and calculate the geometric mean of their weights for the overall final ranking [27]. Ranking is from most stable gene to least stable gene in an ascending order.

## Results

### RNA purity and integrity

All RNA samples included in the present work were within the range of 1.5 and 2.3 ratio of 260/280 value and secondary measures of nucleic acid purity with 260/230 value was equal or above 2 by NanoDrop 2000 (ThermoFisher Scientific). The integrity of each RNA sample considered for RT-qPCR analysis was ensured by reaching a RIN value of 6.5 to 10 (see Additional file 2).

### Expression stability of candidate reference genes

The results obtained by each single algorithm or as comparisons following calculation using the CT values (Additional file 3) with RefFinder are summarized below and listed in detail in Tables 3 and 4.

**Table 3:** **Results of four different algorithms for SPF layer-type chicken tissue samples of healthy birds or those infected with** ***Histomonas meleagridis***

Healthy SPF chickens						Infected with *H. meleagridis*				
Genes	Delta CT	BestKeeper©	NormFinder	GeNorm	RefFinder	Delta CT	BestKeeper©	NormFinder	GeNorm	RefFinder
*RPL13*	*2.49*	*0.73*	*0.745*	*1.302*	*1*	*1.58*	*0.7*	*0.509*	*0.753*	*2*
*TFRC*	*2.61*	*1.24*	*1.192*	*1.302*	*2*	*1.64*	*0.88*	*0.719*	*0.848*	*3*
*TBP*	*2.52*	*1.32*	*0.978*	*1.364*	*3*	*1.52*	*0.82*	*0.377*	*0.753*	*1*
VIM	2.86	1.3	1.406	1.963	4	2.21	1.37	1.733	1.402	5
RPS7	3.14	2.12	2.377	1.626	5	1.87	1.04	1.132	1.112	4
HMBS	3.6	2.27	2.862	2.31	6	2.41	1.43	1.984	1.837	6
HPRT1	3.73	2.52	2.944	2.666	7	2.4	1.92	2	1.634	7
RPLP0	4.96	2.88	4.588	3.239	8	2.61	1.79	2.261	2.03	8

Delta CT compares CT value from two reference genes; BestKeeper© calculates standard deviations (SD) based on raw crossing point; NormFinder gives stability value (Sv) by comparing inter and intra group variations; GeNorm value represents the average expression stability (M); RefFinder compares all other algorithms and gives an overall ranking on the basis of geometric mean.

**Table 4:** **Results of four different algorithms for commercial turkey tissue samples of healthy birds or those infected with** ***Histomonas meleagridis***

Healthy turkeys						Infected with *H. meleagridis*				
Genes	Delta CT	BestKeeper©	NormFinder	GeNorm	RefFinder	Delta CT	BestKeeper©	NormFinder	GeNorm	RefFinder
*TFRC*	*2.39*	*1.22*	*0.908*	*1.333*	*1*	*1.9*	*0.59*	*1.188*	*0.868*	*2*
*RPL13*	*2.41*	*0.91*	*0.923*	*1.333*	*2*	*1.7*	*0.64*	*0.707*	*0.868*	*1*
RPS7	2.41	1.24	0.918	1.45	3	2.2	1.77	1.623	2.033	6
VIM	3.19	1.36	2.466	1.998	4	2.19	1.19	1.653	1.393	3
HMBS	2.99	2.13	2.148	2.466	5	2.23	1.47	1.687	1.872	7
TBP	3.21	2.06	2.488	2.252	6	2.31	1.8	1.826	2.103	8
RPLP0	3.33	3.21	2.711	2.664	7	2.17	1.43	1.635	1.683	4
HPRT1	3.95	2.78	3.45	2.984	8	2.12	1.67	1.501	1.97	5

Delta CT compares CT value from two reference genes; BestKeeper© calculates standard deviations (SD) based on raw crossing point; NormFinder gives stability value (Sv) by comparing inter and intra group variations; GeNorm value represents the average expression stability (M); RefFinder compares all other algorithms and gives an overall ranking on the basis of geometric mean.

### GeNorm

According to GeNorm stability criteria in healthy chicken samples, RPL13 (1.302), TFRC (1.302) and TBP (1.364) were within the threshold range of M value ≤ 1.5 indicating a reliable stability and in case of infected chickens RPL13 (0.753), TBP (0.753), TFRC (0.848) showed even more stable threshold with M values ≤1.0 (Table 3). In turkey samples, RPL13 and TFRC genes demonstrated stability similar to chicken samples with the M values of 1.333 for healthy and 0.868 in infected birds (Table 4).

### NormFinder

Analysis with NormFinder resulted with the same three genes as the most stable ones: TBP, RPL13 and TFRC. The ranking in selected organs of healthy layer chickens was RPL13 (0.745), TBP (0.978) and TFRC (1.192), whereas it was slightly perturbed in tissues from infected chickens, with TBP (0.377) as the most stable followed by RPL13 (0.509) and TFRC (0.719) (Table 3). In turkeys, the ranking was as follows: for samples from healthy birds RPL13 (0.923), TFRC (0.908) and for infected birds RPL13 (0.707) and TFRC (1.188) (Table 4).

### BestKeeper©

BestKeeper© found RPL13 as the most stable gene for both healthy (0.73) and infected layer chickens (0.7) (Table 3). Similar to that, RPL13 (0.91) showed highest stability in healthy turkey tissues also, however, RPL13 (0.64) was ranked behind TFRC (0.59) in samples from turkeys infected with histomonads (Table 4).

### Delta CT

The delta CT results supported GeNorm, NormFinder, BestKeeper© findings. RPL13 (healthy 1; infected 1.58), TFRC (healthy 2.61; infected 1.64), TBP (healthy 2.52; infected 1.52) genes showed the most constant CT values in chicken samples (Table 3). The same trend was found for turkey samples, for which RPL13 (healthy 2.41; infected 1.7) and TFRC (healthy 2.39; infected 1.9) showed lowest variation in CT values (Table 4).

### RefFinder

Conclusive calculations using RefFinder included all above mentioned algorithms: RPL13 (healthy 1; infected 2), TBP (healthy 3; infected 1) and TFRC (healthy 2; infected 3) were most stable in contrast to RPLP0, HPRT1, HMBS which were found to be the least stable genes for healthy and infected layer chickens (Table 3). For turkeys, TFRC (healthy 1; infected 2) and RPL13 (healthy 2; infected 1) showed a consistent stability in both the conditions unlike the remaining reference gene candidates (Table 4).

### Verification of stability for validated reference genes

**Figure 1:**
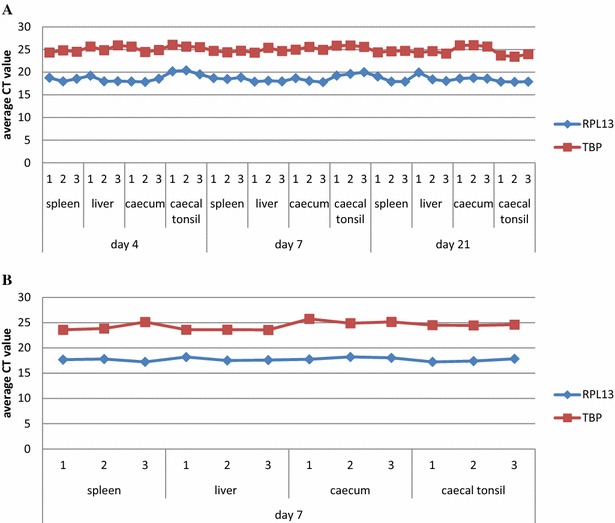
**CT value fluctuations in organs of (A) healthy SPF broiler chickens and (B) birds infected with fowl aviadenovirus.** Graphs were plotted against average raw CT values acquired by RT-qPCR experiment on different tissues of obtained from three SPF broilers (denoted as 1, 2 and 3 in the graph) at different ages. The fluctuation of the CT value was <2.59 for RPL13 and <2.62 for TBP in organs of healthy SPF broilers and <0.98 for RPL13 and <2.1 for TBP in tissues of infected birds.

Additionally, the variation in gene expression of the two most stable genes in layer-type chickens, RPL13 and TBP, was investigated in SPF broiler chickens. The same organs sampled from SPF layer chickens were also investigated from healthy and fowl aviadenovirus-infected SPF broiler chickens (Additional file 3). A stable expression of RPL13 with CT values between 17.8 and 20.39 and TBP with values of 23.43 and 26.05 was noticed in all tissue samples of differently aged healthy SPF broilers (Figure 1A). Following fowl aviadenovirus infection, the CT value was even less diverse, and ranged between 17.24–18.22 for RPL13 and 23.59–25.75 for TBP (Figure 1B).

## Discussion

Recent studies focused on the validation of reference genes applied on different avian tissues [13, 17, 18]. However, so far validation of genes for their stable expression pattern in spleen, liver, caecum, and caecal tonsils of chickens and turkeys in context of an infection with an extracellular pathogen was not performed. Therefore, a wide range of possible physiological conditions (e.g. infections status, age and genetics), which potentially influences the gene expression, were investigated in the present study to identify suitable reference genes.

In a first step candidate reference genes, namely TBP, HPRT1 and HMBS, were selected in the present work according to their previously described stability in gene expression studies of various avian tissues or cells, such as muscular tissues, liver and leukocytes isolated from spleen, thymus and Bursa of Fabricius of chickens [13, 14, 17]. Expression of RPS7 together with TFRC was described in chicken and turkey brain tissue [18]. In addition to the above mentioned genes, VIM, RPL13 and RPLP0 were also validated in the present analysis. VIM and RPL13 were not found to be stable in a study evaluating pan-avian reference genes on brain samples [18] and RPLP0 was only analyzed in organs of mammals [28]. Anyhow, they were included in the current study to broaden the spectrum of metabolic and structural pathways. Other previously reported reference genes like 18S and 28S rRNA or GAPDH were not validated in the present work due to certain aspects that makes them unlikely to be used for normalization: the expression of 18S and 28S rRNA is regulated by the RNA polymerase I enzyme, whereas the synthesis of mRNAs to be measured is processed by the RNA polymerase II [6, 29]. Furthermore, both genes do not harbour any introns, which implicates that the amplification of genomic DNA, if not removed properly, is possible. In agreement with that, Olias et al. [18], who performed a multi avian species study, also recommended to avoid 18S rRNA as a reference gene, despite of the proven stability. GAPDH, on the other hand, is involved in the glycolytic pathway and its expression depends on the respective tissue and different experimental conditions such as glucose deprivation or stress. Therefore, also GAPDH was described to be unsuitable for normalization of RT-qPCR experiments [6, 29].

According to the MIQE guidelines reference genes can vary between different species and have to be validated even between closely related species [5]. Consequently, the expression of all reference candidates of this study was investigated separately in chickens and turkeys. Furthermore, genetic variations as well as previously demonstrated immunological differences between SPF layer- and SPF broiler chickens [30], which potentially affects the gene expression, were considered in the present work. Previous studies also described different expression patterns of reference gens according to the type of organ [13, 17, 18]. This prompted us to include the immune organs, spleen and caecal tonsils, together with the liver and the caecum, organs that are fundamental in the metabolism of birds. Age dependent changes in gene expression were covered by using SPF layer chickens and turkeys from day-old to 49^th^ day of life, and SPF broiler chickens from day-old to 21^st^ day of life. Finally, the impact of different kind of pathogens on the gene expression in organs of host birds was investigated. For that, chickens and turkeys were infected with the extracellular pathogen *H. meleagridis* following a well-defined infection model [31]. Hence, reference gene candidates in inflamed and immune organs of birds at different time points following infection were included. Accordingly, the most suitable reference gene candidates were used in organ samples from SPF broiler chickens infected with fowl aviadenovirus to validate the stability following infection with an intracellular pathogen.

The expression of each reference gene for every sample was analyzed with different algorithms: GeNorm, NormFinder, BestKeeper© and delta CT. The rankings of assayed genes were not always identical due to variations in statistical calculations by different algorithms, a phenomenon recently reported [17]. Therefore, it was crucial to get a consensus in the outcome of the applied algorithms, for which purpose the RefFinder software was applied. Overall, it was found that RPL13 gene was the most stable expressed gene in the examined tissues of SPF layer chickens regardless of an infection with *H. meleagridis*. TBP and TFRC were both shown to be stable as well; however, there were slightly higher variations in the expression levels of TFRC in tissues of infected SPF layer chickens. Depending on the experimental setup TFRC also can be used as reference gene along with RPL13 and TBP for chickens. All other reference gene candidates calculated with RefFinder were not considered as stable for SPF layer chickens due to severe variations in their expression profiles. Hence, RPL13 and TBP were further investigated in tissues of non-infected and infected SPF broiler chickens with fowl aviadenovirus. These additional investigations confirmed the stable expression of both genes in SPF broiler chicken spleen, liver, caecum and caecal tonsil samples. Our findings demonstrated that neither the genetic background of chickens nor the nature of an infectious agent caused remarkable variations of RPL13 and TBP expression in spleen, liver, caecum and caecal tonsil, suggesting them as optimal candidates for normalization of RT-qPCR experiments. Similar to the results found in chicken tissues, RPL13 was also determined to be the most stable gene, followed by TFRC in spleen, liver, caecum and caecal tonsil samples from healthy and infected turkeys with the extracellular pathogen. In contrast to data from chickens, presented here and elsewhere [13, 14], TBP of turkeys ranked on the eight position using RefFinder, which indicates the heterogeneity in the expression of certain candidate reference genes between gallinaceous species. Thus, our findings also show the possibility of a variation of reference genes expression between different bird species, even if they are closely related. On the other hand, TFRC was stable in both poultry species which is in the agreement with previous findings investigating brain tissue [18].

According to the MIQE guidelines, a reference gene needs to be validated and established for every species and for different physiological conditions. The guidelines furthermore specify verification of a used reference gene for every experimental settings, otherwise the variation of reference genes expression may adversely affect the results and with it the biological conclusion. The RPL13 and the TBP genes of chickens and the RPL13 and the TFRC genes of turkeys were shown to be highly stable in the present experimental setting and are therefore recommended to be first priority candidates for gene expression studies in case spleen, liver, caecum and caecal tonsil tissues are targeted.

## Additional files

**Additional file 1.** **Candidate reference genes efficiency.** Optimized concentrations of all candidate reference genes primers and probes are given with efficiency value for chicken and turkey species.

**Additional file 2.** **RNA integrity number measured with Bioanalyzer2100.** The RIN values of all the samples used in the experiment are given.

**Additional file 3.** **Raw CT values obtained in the present work.** The raw CT values of all RT-qPCR experiments were used for calculations with different algorithms to assess the stability of each reference gene candidate.
