# Supplementary material for: The 60S ribosomal protein L13 is the most preferable reference gene to investigate gene expression in selected organs from turkeys and chickens, in context of different infection models
Source: Vet Res. 2016 Oct 20;47:105. doi: 10.1186/s13567-016-0388-z (PMC5073923; doi:10.1186/s13567-016-0388-z)
Supplement: Supplementary file 1 — Additional file 1. Candidate reference genes efficiency. Optimized concentrations of all candidate reference genes primers and probes are given with efficiency value for chicken and turkey species. [file 13567_2016_388_MOESM1_ESM.docx]

| gene^1^ | primer concentrations for chicken  (nM) | efficiency for chicken  (%) | primer concentrations for turkey  (nM) | efficiency for turkey  (%) |
| --- | --- | --- | --- | --- |
| **TFRC** | 400 | 98 | 400 | 100.9 |
| **TBP** | 600 | 100.4 | 600 | 99.2 |
| **HPRT1** | 700 | 97.3 | 700 | 98.2 |
| **VIM** | 400 | 96.4 | 500 | 97.8 |
| **RPS7** | 400 | 97.3 | 400 | 95.2 |
| **RPL13** | 500 | 95.1 | 300 | 96 |
| **HMBS** | 400 | 100.3 | 400 | 98.7 |
| **RPLP0** | 600 | 96.4 | 600 | 96.8 |

^1^ For every candidate gene, 100nM of probe concentration was used for both the species. Concentrations of primers were different for chickens and turkeys.
